# Investigating local policy drivers for alcohol harm prevention: a comparative case study of two local authorities in England

**DOI:** 10.1186/s12889-017-4841-3

**Published:** 2017-10-18

**Authors:** John D. Mooney, John Holmes, Lucy Gavens, Frank de Vocht, Matt Hickman, Karen Lock, Alan Brennan

**Affiliations:** 1 Faculty of Health Sciences and Well-being, University of Sunderland, City Campus, Dale Building, Sunderland, SR1 3SD UK; 2 School of Health and Related Research, University of Sheffield, Regent Court, Regent Street, S1 4DA, Sheffield, UK; 3 School of Social and Community Medicine, University of Bristol, Canynge Hall, Whatley Road, Bristol, BS8 2PS UK; 4 London School of Hygiene & Tropical Medicine, Tavistock Place, London, WC1H 9SH UK

**Keywords:** Alcohol policy, Local government, Policy prioritization

## Abstract

**Background:**

The considerable challenges associated with implementing national level alcohol policies have encouraged a renewed focus on the prospects for local-level policies in the UK and elsewhere. We adopted a case study approach to identify the major characteristics and drivers of differences in the patterns of local alcohol policies and services in two contrasting local authority (LA) areas in England.

**Methods:**

Data were collected via thirteen semi-structured interviews with key informants (including public health, licensing and trading standards) and documentary analysis, including harm reduction strategies and statements of licensing policy. A two-stage thematic analysis was used to categorize all relevant statements into seven over-arching themes, by which document sources were then also analysed.

**Results:**

Three of the seven over-arching themes (drink environment, treatment services and barriers and facilitators), provided for the most explanatory detail informing the contrasting policy responses of the two LAs: LA1 pursued a risk-informed strategy via a specialist police team working proactively with problem premises and screening systematically to identify riskier drinking. LA2 adopted a more upstream regulatory approach around restrictions on availability with less emphasis on co-ordinated screening and treatment measures.

**Conclusion:**

New powers over alcohol policy for LAs in England can produce markedly different policies for reducing alcohol-related harm. These difference are rooted in economic, opportunistic, organisational and personnel factors particular to the LAs themselves and may lead to closely tailored solutions in some policy areas and poorer co-ordination and attention in others.

## Key message

Prioritisation of alcohol harm prevention policies can vary substantially between English local authorities due to differences in local circumstances and conditions. Awareness of how these differences can arise may help guard against imbalances in strategy.

## Background

The often considerable political challenges inherent in pursuing national level public health policies to reduce alcohol harm has prompted policy makers in a number of countries to explore locally tailored approaches [1–3]. Such measures have particular relevance in England following two recent policy shifts: Firstly, the transfer of public health teams from the National Health Service to 152 upper tier local authorities (LAs) and, secondly, the designation of local Directors of Public Health as responsible authorities able to challenge applications for alcohol retail licenses [4]. These changes offer considerable scope for intervention as they effectively serve to co-locate within LAs increased powers to regulate alcohol availability alongside commissioning responsibilities for alcohol treatment and early intervention services [5]. Their scope for radical policy formulation however is potentially constrained by competing concerns, most notably the need to foster a vibrant local economy [6]. For many post-industrial UK towns and cities where development of a night-time economy played a key role in urban regeneration, a tension may be present between regulating and supporting business for whom alcohol sales play a major role [6].

The response of LAs to their new statutory responsibilities around alcohol policy has been highly variable and strongly informed by their differential prioritisation of the immediate socially disruptive effects of alcohol and its longer term chronic health impacts [7]. Research to date suggests a tendency for decisions to be informed by local experiential evidence rather than formal evidence sources such as peer reviewed studies or external expertise [8]. Although the variability of LAs’ new approaches to alcohol policy has been noted, the processes which drive this variability are less well explored [6, 7]. This includes how particular policies and combinations (or suites) of policies are chosen and how decision-making is variously informed by the identified or perceived needs of their local populations, the prioritisation of alcohol-related harm as well as resource constraints and competing priorities.

Within the context of this new policy environment, this paper aims to identify and examine the most significant policy drivers that have led to different suites of alcohol policies being adopted in two LAs in Northern England. Using a qualitative comparative case study approach, involving interviews with key informants and documentary analysis, we seek to understand the considerations that have informed differences in policy and strategy around alcohol licensing and availability, as well as approaches to the provision and availability of targeted screening and brief intervention programmes.

## Methods

Case study research is a qualitative approach of particular value for developing a rich understanding of deliberately selected exemplars of a phenomenon [9]. Data can come from a range of sources and, in this case, has been drawn from interviews with key informants and supplementary documentary analysis. Case study LAs were selected on two main criteria. First, high levels of alcohol-related harm as indicated by their local alcohol profile, a characterisation of alcohol-related harm levels within each English LA [10]. This suggested the case study sites would be likely to give significant priority to reducing alcohol-related harm. Second, information already in the public domain (from local news articles and a national level needs assessment), indicated differences in their overall approach, particularly in their degree of regulation of the night-time economy and how they perceived their role in the identification and targeting of those already at elevated risk of alcohol harm. The selection of two cases with particularly contrasting approaches was intentional in order to generate insights into how locally-specific factors and conditions  might shape differences in policy decisions. Thus, the two LA case study sites were:

- LA1: A post-industrial and ethnically diverse city with high rates of alcohol-related hospital admissions. The city has high levels of deprivation and limited employment opportunities for professionals. Prior to this research, there was significant investment by the city in dedicated alcohol treatment services. A relatively widely dispersed population and proximity to another larger urban conurbation has contributed to a limited ‘night-time economy’.
- LA2: A large post-industrial city with a vibrant night time economy serving an economically diverse population. Premature mortality rates were high, particularly from liver disease and the city had developed and pioneered significant local initiatives to tackle alcohol-related crime and disorder, primarily around ‘on-trade’ restrictions on the types of premises associated with such disorder.

Based on previous ethnographic studies of local alcohol policy decision-making processes [8, 11], available guidance documents and supplementary informal advice from colleagues working in local government, we identified the three core key informants to interview as: (i) Public Health; (ii) Licensing/Trading standards; and (iii) Commissioning. Further participants with expertise on a particular policy, process or intervention (e.g. specialist police licensing officers; clinical providers) were identified through snowballing. Table 1 shows the number of interviews by role in each LA.

**Table 1:** Interviewees from each case study Local Authority

Interviewee Role	LA1	LA2
Police	1	1
Licensing/Trading standards	1	2
Public Health	1	1
Commissioning	2	1
Treatment Services/Clinical	1	1
Information analyst	0	1
TOTAL	6	7

Semi-structured interviews followed a topic guide which focused on the characteristics and development processes through which the current mix of alcohol harm prevention policy had come about. Advice was sought from two alcohol policy leads in separate local authorities (outside of the case study areas selected for this study) and with whom the topic guides were piloted. Included questions related to the respondents perceived level of importance which  they attributed to the prioritisation of reducing alcohol-related harm, the LA’s general strategic approach within a local context and what had worked well and not so well. Interviewees were invited by email for a 1-h semi-structured interview [12], although interview length ranged between 26 and 93 min. Eleven of the thirteen interviews were conducted face to face and two by telephone. While interviews sought to cover a broad range of local alcohol policies and programmes, interviewees were free to expand on those aspects most relevant to their own area of experience or expertise.

Interviews were recorded and transcribed verbatim. Thematic analysis was used with a particular focus on (1) identifying key themes informing and driving the development of local alcohol policies and (2) addessing the proposition that recent policy changes described above may facilitate the tailoring  of approaches to tackling alcohol-related harm to local contexts. Initial primary coding was undertaken by the first author with all statements of relevance to the research objectives assigned a primary code. Primary codes were then grouped into secondary overarching themes which were discussed and agreed with collaborators on the broader project from which this paper emerged. NVivo version 10 for windows (QSR International) was used to analyse transcripts.

The documentary sources used were the current statement of licensing policy (a statutory requirement for each LA), along with their respective alcohol harm reduction strategies. Other significant documentary sources for background context and between LA comparisons were the Alcohol Needs Assessment Research Project (England) [13]; a third sector published report highlighting some examples of good practice (not cited for disclosure protection) and Home Office Licensing Statistics [14] which provided summary totals for licensing applications, challenges, outcomes and appeals. The needs assessment and the third sector report were independently highlighted by at least two interview respondents when prompted about other publications which covered their strategic approaches. Documentary sources were thematically analysed using the agreed secondary themes derived from interview primary codes.

## Results

The two participating LAs were situated in cities of comparable population size: LA1 was significantly further south than LA2 (though not in the south of England) and was less centralised around a large city centre. Both were three hours or more travelling time by train from London and both were university cities within commuting distance of academic departments with significant interests in alcohol related research. Both case study LAs also cited alcohol and drug misuse within their top three public health priorities within their respective community safety strategies. From the thematic analysis of interview transcripts, seven second level themes were identified: drinking environment; treatment and intervention; available evidence; planning and strategy; public health burden; targeting risk groups and wider impact. Given the predominant focus in one LA on upstream interventions around the drink environment and availability, contrasting with a focus of the other LA on treatment approaches and risk group targeting, we chose to structure our findings around just three over-arching themes: namely (1) drinking environment, (2) treatment and intervention and (3) barriers and facilitators, which were materially relevant to both these approaches. The third newly introduced over-arching theme subsumed the remaining five themes in that their component topics effectively served either as barriers or facilitators. The three over-arching or ‘third level themes’ therefore provided for the greatest contrast between the comparison LAs, as well as pertaining directly to the overall aims of the study.

### Drinking environment

LA1 and LA2 contrasted in their approach to the drinking environment with LA1 taking a largely non-regulatory approach and focusing on negotiated agreements while LA2 focused efforts more on proactively exercising its regulatory powers.

The night-time economy in LA1 is policed by a small team of specialist officers working closely with local alcohol retail licence holders. Although a formal police objection is raised by default on all new licence applications, this tactic is used to encourage applicants to work with the police and respond to their concerns by incorporating modifications to their licensing schedule. The objection is then withdrawn if the police licensing team are satisfied with the applicant’s responses. The specialist (police) licensing officers then follow up on implemenation by licensees  by monitoring their promotional activities on social media and facilitating agreements that they (licensees) will refrain from undercutting each other (in a manner which leads to irresponsible price wars that may compromise licensing objectives):

*…although there are no ‘all inclusive deals’ now, they still stick to ‘gentleman’s agreement’ arrangements that they won’t (for example) go under £1.20/£1.30 for a bottle of beer [LA1; Police].*

LA1 also refrained from designating any local areas as cumulative impact zones (CIZs, also referred to as cumulative impacts policies or CIPs), an action that means any new license application will only be accepted in the designated area if the applicant demonstrates they will not they will not exacerbate existing alcohol-related problems. This represents a change from the standard burden of proof whereby license applications can only be rejected if public bodies demonstrate that existing problems will be exacerbated or more specifically, that the licensing objectives are likely to be compromised. From the police’s perspective in LA1, CIZs are seen as less preferable to working with license applicants to address any concerns:

*…So you can agree relatively stringent operating conditions (by negotiation) on licences even though we don’t have cumulative impact [LA1; Police].*

Reluctance to use CIPs also reflected concerns about discouraging economic activity:

*…the trading sector don’t generally like those (CIPs), because they are perceived as limiting to local business investment and the local economy [LA1; Trading Standards].*

In contrast, the local authority in LA2 have pursued a more regulatory approach rooted in a desire to challenge the city’s reputation for an unruly nightlife:

*there (was) a view in this city which I think is now diminishing a little bit that our late night economy or image as a ‘party city’ were out of control [LA2; Licensing].*

Thus, LA2 fully utilised  their regulatory powers to implement cumulative impact policies and a *‘*late night levy’ (which imposes a supplementary annual charge on premises selling alcohol between midnight and 6 am) [15]. Although subject to legal challenge, the resulting regulatory approach had some support among license holders who saw the benefits, such as the increased police presence, especially once it was appreciated that the financial costs to businesses of the regulatory regime were being reinvested  and used to manage the night-time economy:

*as long as the licensees see transparency between collection and spend they haven’t got that much of a problem really and that’s where we are with it [LA2; Licensing].*

LA2 had also allocated a 30% reduction in the fee for premises which sign up to their "business best practice" scheme which echoes LA1’s approach in establishing good working relationships with license holders to address police concerns and deliver a particular vision for the city centre. For example, locally negotiated licensing restrictions, initially proposed by a new license applicant in a CIZ area, are now voluntarily written into the licenses of eleven city centre premises. Both the police and local council regard this as consistent with their objective of achieving a more ‘upmarket’ and diverse night time economy:

*…it’s all about trying to drive up the quality and the diversity of the offer [LA2; Licensing and also highlighted by Police].*

The first impression of LA1’s approach to minimizing any adverse impact of alcohol misuse might therefore be seen as more ‘lighter touch” that than of LA2, with an apparent reluctance to place area based statutory restrictions on commercial alcohol trading. On closer reflection, the less formal regulatory environment of LA1 however was not without relatively well developed local procedures for the close scrutiny of licensing arrangements, dependent on specialist police officers and the cultivation of personal relationships with licensees and other stakeholders:

*I tend to I work with everybody be it solicitors, licence trade consultants or the premises licence holders/the owners the door staff and everybody …and if they have got a problem I put them on an action plan so it’s all highlighted [LA1: Police]*.

### Treatment and Intervention

While LA1 and LA2 both had well-established, but different, approaches to reducing acute alcohol problems arising from the licensed trade, only LA1 had a clear strategy for addressing chronic problems within healthcare settings. Investment in large-scale screening and brief intervention programmes has been a major component of LA1’s approach to tackling the harm due to alcohol since an Alcohol Health Needs Assessment undertaken in 2010. This had played a key role in crystallising and evidencing concerns about drinkers from minority ethnic groups and the need for an integrated approach across council departments:

*Anecdotally we’ve known there is an issue with alcohol in the South-East Asian community and following on from work undertaken as part of the needs assessment, we’ve been able to engage community leaders such as Imams [LA1; Public Health]*

*The comprehensive needs assessment also helped identify defined population sub-groups for whom drinking was associated with adverse life circumstances, such as unemployment, insecure residency status, enabling a joint services approach to treatment provision and not tackling drinking in isolation [LA1; Commissioning]*

Indeed LA1 informants were quite explicit about the extent to which the large scale health needs assessment (HNA) helped to make the case for investment in screening and early intervention

*…the health needs assessment was a massive boon and the start of being able to demonstrate what the likely benefits of investment would be, which persuaded the powers that be [LA1; Public Health].*

This situation can be contrasted with LA2 in which there was no reference to any needs assessments and as the harm reduction strategy points out, at the time of publication, a comprehensive needs assessment focused on alcohol misuse had not been undertaken:

*…there has not been a needs assessment to inform how best to adopt a preventative or treatment-based approach particularly for binge drinkers and those drinking excessively at home [LA2; Alcohol Harm Reduction Strategy].*

Financial considerations were also prominent for the clinical commissioning group^1^ (CCG) in LA1 who were concerned about the resource implications of high numbers of alcohol-related hospital admissions:

*..(the) longer term objective is to reduce the numbers of people that need to come through to expensive in-patient detox services [LA1; Commissioning].*

The response to these concerns has been two fold and has involved a community delivered programme of screening and brief interventions, which has included extensive training and awareness-raising for all front-line health care professionals and a two year pilot of alcohol specialist nurses based within secondary care entirely funded by the CCG.

Within LA2, there has been less co-ordination in the roll-out of treatment access and brief intervention programmes and this was recognised in the local alcohol harm reduction strategy as well as in a national Alcohol Needs Assessment Report [13]. A needs assessment, which was influential in LA1, had not been undertaken in LA2 at the time of data collection and there was a narrower long-term focus on the visible, acute and social order consequences of excess alcohol consumption in the night time economy. This was reflected in performance monitoring priorities:

*Public health are conscious of hospital admission indicator, NI39 (the only one that people ever look at), but at present nobody is really monitoring it but, in the new set of core performance indicators, currently under development, we will include NI39, although it is no longer a statutory requirement [LA2; Public Health].*

A number of local initiatives are currently being evaluated including the location of specialist alcohol liaison nurses in secondary care, although these are mainly targeted at heavier drinkers as a result of the focus on ‘intoxicated episodes’ requiring hospital attendance:

*Yes I mean they’re looking at very much (those patients) who have been red-flagged as high risk, very high risk or dependent drinkers [LA2: Treatment Services].*

One hospital trust within LA2 had also now initiated a broader screening policy where a form of ABI is included as standard in the pre-op assessment:

*so they’ve taken on doing ABI with all of the patients that they see prior to surgical intervention, when they’re having a pre-assessment. [LA2: Treatment Services].*

For primary care settings in LA2, it was acknowledged that there was scope for more widespread implementation of screening and brief interventions and that current localised pilot programmes based on different provider set-ups, will help to identify and clarify the best model to roll out:

*…it has happened for some time on an ad-hoc basis (so although) many practices are already doing it, it hasn’t been implemented in a strategic way [LA2: Treatment Services].*

### Barriers & Facilitators

In addition to the apparently contrasting LA policy responses towards tackling alcohol harm between tighter regulation of the drinking environment on one hand and treatment based approaches on the other, a range of local contextual factors also acted as barriers or facilitators to the resulting strategic focus of alcohol harm reduction efforts in each LA. Table 2 summarises the most significant policy drivers cited in this and previous sections, which, on the basis of interviewee comments and documentary sources, would appear to have had the greatest influence.

**Table 2:** Summary of identified policy drivers from interviews & document sources (Number of times independently referred to by an interview or document source in parenthesis – see abbreviation key below for source initials)

LA SITE 1		LA SITE 2	
Barriers	Facilitators	Barriers	Facilitators
Resource constraints leading to a decision to focus on over-riding priorities and limited local police resources (×3: PC; PH; CM).	Comprehensive Health Needs Assessment which identified unmet needs in defined sub-populations (×3: CM; PH; DC).	Large metropolitan area leading to problems planning ‘joined up’ services in providing treatment options and pathways (x2: CT; PH).	Successful application for extra funds specifically for tackling alcohol fuelled violence and disorder (×4: PH; LT; PC; DC)
Not wanting to discourage commerce (×2 PH; LT).	Alcohol commissioning co-located with Public Health within organisational structure (×2: CM; PH).	No comprehensive area alcohol needs assessment (×2: PH; DC).	Access to specialist legal expertise making legal team less risk averse about implementing novel policy measures likely to be challenged by the industry (×3: PH; LT; PC)
Information sharing difficulties/IT compatibility issues (×4: CM; PH; IT; TP).	Informal close working: police & licensees (×2: PC; LT).	Under-provision and patchwork nature of alcohol specialist treatment services (×3: CL; CM; DC).	Pro-active police around licensing, strongly motivated to tackle poor public image of the city in relation to drinking (×3: PC; LT; DC)
Necessity of tackling high admissions – restricting capacity for a wider approach (×3 PH; CM).	Enlightened CCG willing to fund Hospital ABIs (×4: CM; CT; PH; DC)	Little apparent engagement from CCGs (×2: CT; TP).	Capacity to diversify large and vibrant night-time economy (×3: LT; PC; TP).

*Abbreviation: PC* Police, *PH* Public Health, *CM* Commissioning, *CL* Clinical/treatment role, *LT* licensing/trading standards, *IT* information specialist, *TP* Third party interviews, *DC* document source

Aside from the financial support from the CCG in LA1 for a comprehensive alcohol treatment pilot, referred to under Treatment and Intervention, the location of the commissioning team within public health was believed to be a significant structural enabler of commissioning well evidenced interventions such as ABI’s:

*“We have brought the alcohol [treatment] commissioning team into public health as part of the department – (which) has been a big thing as well they work with us and take the lead on lots of commissioning projects” [LA1; Public Health]*

The comprehensively commissioned treatment approach adopted in LA1 was also underlined by an influential senior general practitioner and member of the local CCG who sought to raise awareness around the well evidenced value and cost effectiveness of ABIs in primary care:

*Our clinical lead for alcohol is a local GP. And he's great. You know, really, really interested in pushing the (ABI) agenda and encouraging wider provision and take-up… [LA1; Commissioning].*

Whereas dedicated extra resources were available in LA1 for the treatment pilot, LA2 benefited from being awarded additional funding to reduce alcohol fuelled disorder:

*[The funding] helped. [The funding]…got recognition. It wasn’t just about recognition, but I think that brought about a lot of communal working, which I think previously there wouldn’t have been funding to do… [LA1; Commissioning].*

From an organisational perspective in LA2, close collaborative working arrangements between the police and the LA were believed to be strongly facilitated by the council’s statement of licensing policy and the level of legal expertise they were able to rely upon to deal with objections to proposed policy measures such as the late night levy:

*the council’s statement of licensing policy is an outstanding document which assists us hugely in being able to effectively deal with two things in particular: one being the control and the regulation of the existing night time economy and two being restricting the proliferation of retail outlets for alcohol off licences [LA2: Police]*

*..while there were many in the trade who were trying to prevent it, the skill of the legal team was such that they were unable to do so [LA2: Licensing].*

Perhaps unsurprisingly, practical considerations around aspects of resources and infrastructure have had a significant influence on aspects of responding efficiently to stated alcohol harm priorities. CCG funding for ABI interventions for instance in LA1, contrasts with no such commitments in LA2, resulting in a more sporadic set-up:

*Some hospital trusts are doing it (ABIs) and financing it themselves – so no resources forthcoming at present from CCG or LA. [LA2: Treatment Services].*

On the issue of infrastructure, LA1’s capacity for monitoring incidents and harms through routine data sources and linking them up in a meaningful way was adversely impacted by deficiencies in their information sharing systems:

*One of the biggest barriers has in fact been IT issues (which) hamper real-time recording/data- sharing efforts (around alcohol) – an adequate IT infra-structure is definitely lacking – often means going to lots of different systems to pull out related information such as localised health and incident data... [LA1; Public Health (also highlighted by Commissioning)].*

While genuine population health needs analysis alongside the likely acceptability of regulatory interventions have therefore together informed the different emphases in policy responses, there are clearly a wide range of LA specific organisational enabling or discouraging factors, that can influence policy choices.

## Discussion

The two case study LAs expressed similar levels of perceived commitment to reducing alcohol harm and both clearly saw alcohol as a public health priority; however, this commitment translated into very contrasting approaches to alcohol policy. LA1 exhibited a strategy targeting risk-premises and risk groups which addressed both licensing and healthcare interventions, with the former focusing on negotiated relationships with license holders and applicants. In contrast, LA2’s response was characterised by a less well developed programme of access to screening and healthcare interventions, alongside a substantive suite of regulatory measures aimed at transforming the night-time economy. These were not absolute or static differences (e.g. LA1 did establish working relationships with licensees and LA2 commissioned healthcare interventions in a fragmented fashion while anticipating more work in this area in future), but they served to highlight clear differences in concerns, priorities and resource availability. These differences appeared rooted in four factors: (i) differences in the relative importance and profile of the respective night-time economies; (ii) organisational/structural components, such as the proximity of public health with treatment commissioning (co-located in LA1); (iii) the availability of dedicated additional resources (in LA1 for treatment/screening by the CCG and in LA2 from a charitable fund for tackling crime and disorder) and the ready availability of specialist advice (legal expertise in LA2) or clinical champions (e.g. proactive clinicians in LA1, with a significant interest in alcohol treatment).

A major strength of the case study in descriptive exploratory research is its capacity to draw upon and compare content and emergent findings from a range of different sources [9]. The findings above are strengthened by using extensive cross-corroboration between interviewees, documentary sources and independent third party interviews (see Table 2). This minimises, although does not eliminate, the possibility that interviewees perspectives are selective and based on individual beliefs or preferences, a risk that could have been exacerbated by the relatively low numbers of informants in each LA. Although pro-actively seeking divergent views is an established strategy for validating the coherence of higher order themes in qualitative research [16], the high degree of convergence (as evident in Table 2) and the ability to explain differing emphasis through appreciating participants' different professional perspectives, indicated early on in this study that such an approach would be unlikely to add any further useful insights.

Although the comparison of two case study sites does not provide a generalisable picture of contemporary local authority-level alcohol policy in England and may therefore be seen as a limitation of the current  approach, it does facilitate a focus in this instance on contrasting strategic priorities with regard to reducing alcohol harm and the processes and factors which can contribute to that. Of course, the restriction to just two sites in this study and at one particular point in time, can only hint at the complexities involved in the development of local policy responses to alcohol and we would see this environment as a perfect illustration of recent calls for a ‘complex systems approach’ in the development and appraisal of solutions to modern public health problems [17]. Studies with larger numbers of LAs are likely to lend themselves well to such approaches using established socio-ecological frameworks such as the ANGELO framework used in obesity policy analysis [18], which would encompass the different ‘policy domains’ that impact on alcohol at both macro and micro-level settings.

The capacity for local government to develop locally responsive alcohol harm reduction measures has received renewed attention in recent years, which in the UK has been facilitated by changes to the public health and licensing infrastructure [6, 19]. Internationally also, there is recognition that locally derived policies benefit from a democratic legitimacy when supported by local populations [20] and their dispersion and variability present a challenge for alcohol industry efforts to influence policy [21] (although legal challenges against licensing regulation are common). The results above highlight both that localism in alcohol policy can produce responses tailored to local contexts (e.g. utilising the strong legal team in LA2 to take advantage of regulatory powers and focusing on challenges particular to minority ethnic groups in LA2), but also that uneven strategic responses to alcohol policy can emerge (e.g. some local authorities not having the legal resources available to LA2 and LA2 also being slower to develop a comprehensive alcohol-related healthcare policy and sevice model). Given substantially different starting points in terms of their respective alcohol harm profile, it is hazardous to compare changes over time between the two case study LAs, although LA2, which emphasised availability and the drinking environment, has seen marginally greater reductions in morbidity and mortality indicators over the course of the study and since its completion.

The importance of organisational factors in the viability of and access to alcohol treatment services has previously been highlighted from both a management perspective [22] and that of service users [23]. Concerns have also been expressed that a form of the “inverse care law” may be emerging in England that is partly attributable to the recent structural changes in the commissioning of preventative public health services [24]. LA2’s intentions and published plans to improve its alcohol-related healthcare services may suggest it is simply at an earlier stage in the implementation process described by Simpson [22]. A key implication of our findings therefore is that from the point of view of individual LAs, support for a broad-based and comprehensive LA policy response aimed at redressing alcohol health harms is to be recommended, whatever the focus of their current priorities. Encouraging CCGs such as that in LA1 for example, to publicise the benefits of investment in treatment services, at a time when many may not see the benefits, is also likely to help foster an understanding of the wider advantages of maintaining or increasing such investments.

Recent encouraging findings on the likely beneficial impact of more pro-active regulatory policy approaches on the part of LAs on alcohol harm statistics such as hospital admission rates [25] and on the more traditional indices of crime and disorder [26], help strengthen the evidence case for public health teams in particular to make good use of their recently acquired ‘responsible authority’ status. Experience of the more pioneering LAs in this area also highlights that the way policies such as cumulative impact zones [27] and reducing the availability of cheap high alcohol content beverages [28] are implemented are likely to exhibit considerable variability according to local circumstances. A degree of heterogeneity therefore in LA alcohol policy responses and structures is therefore to be expected and it is unsurprising that this extends across the full spectrum of LA influence on treatment and prevention. While the current study has documented some of the more significant barriers and facilitators that might influence the particular combination of policies seen in any particular LA, it is clear that the prospects for maintaining a comprehensive response will be enhanced by ensuring an appropriate mix of treatment and prevention approaches and by spreading effective innovations and good practice between LAs.

## Conclusions

New powers over alcohol policy for LAs in England can produce markedly different local policy mixes for reducing alcohol-related harm. These differences are rooted in economic, organisational and personal factors particular to the LAs concerned and may lead to closely tailored solutions in some policy areas with less than may be optimal attention paid to others. Those working in public health need to be vigilant of where and how these imbalances might arise, so that they can work towards proactively addressing them.

## Footnotes

1 Clinical Commissioning Groups are collaborative local groups of primary care practices who in England now hold the health service budgets for their local populations.
